# Effect of 8‐week leucine supplementation and resistance exercise training on muscle hypertrophy and satellite cell activation in rats

**DOI:** 10.14814/phy2.13725

**Published:** 2018-06-27

**Authors:** Chang Hyun Lim, Ju Hyun Gil, Helong Quan, Dang Ha Viet, Chang Keun Kim

**Affiliations:** ^1^ Human physiology, Korea National Sport University Seoul Korea; ^2^ Exercise and Metabolism Research Center Zhejiang Normal University Jinhua China; ^3^ College of Physical Education and Health Science Zhejiang Normal University Jinhua China; ^4^ Sport Science and Technology Institute Hochiminh City University of Sport Hochiminh City Vietnam

**Keywords:** Embryonic myosin, Leucine, Resistance exercise, Satellite cell

## Abstract

We investigated the effects of regular leucine intake and/or resistance exercise training on skeletal muscle hypertrophy and satellite cell activity after the administration of different doses of leucine. Ten‐week‐old Sprague–Dawley rats were assigned to six groups (*n* = 7 per group): a control group (Con), two groups receiving either 10% (0.135 g/kg.wt) (Leu10) or 50% (0.675 g/kg.wt) (Leu50) leucine supplementation, and three exercise groups receiving 0% (Ex), 10% (Leu10Ex), and 50% (Leu50Ex) leucine supplementation. The rats performed ladder climbing exercises thrice per week for 8 weeks, and received leucine supplements at the same time daily. Muscle phenotypes were assessed by immunohistochemistry. MyoD, myogenin, and IGF1 protein levels were determined by western blot. The Leu50Ex group displayed significantly higher numbers of positive embryonic myosin fibers (0.35 ± 0.08, 250%) and myonuclei (3.29 ± 0.3, 118.7%) than all other groups. And exercise training groups increased the cross‐sectional area, the number of satellite cells and protein expression of MyoD, myogenin, and IGF1alpha relative to the Control group (*P *<* *0.05). However, Only leucine supplementation group did not increase skeletal muscle hypertrophy and satellite cell activity, regardless of the dose (*P *>* *0.05). Leucine intake accompanied by regular exercise training may increase satellite cell activation in skeletal muscles, and improve muscle quality more effectively than continuous leucine ingestion alone.

## Introduction

The maintenance of an adequate muscle mass not only enables an independent life, with normal functioning muscles, but also improves the quality of life by lowering the incidence of metabolic disorders such as obesity, hypertension, and type II diabetes (Clynes et al. 2015). Muscle mass is controlled by the balance between protein synthesis and proteolysis. Regular physical activity and adequate nutrition can increase protein synthesis, and subsequently, muscle mass. Resistance training is the most effective method of exercise for increasing protein synthesis in the skeletal muscle. Resistance training can activate the expression of mRNAs associated with protein synthesis, such as the mammalian target of rapamycin‐ (mTOR) and mitogen‐activated protein kinase (MAPK), and activate protein signaling pathways via a single stimulus (Dreyer et al. 2006). Regular resistance training not only induces the phosphorylation of proteins involved in the signaling pathways underlying muscle synthesis, such as mTOR and MAPK, but increases the activity of satellite cells, which are muscle stem cells, to promote muscle repair, regeneration, and growth (Kadi and Thornell 2000); it is therefore known as the most effective method of exercise for improving the cross‐sectional area of muscle fibers, muscle strength, and muscle function (Kosek et al. 2006).

As the importance of the nutrient intake for improving athletic performance and maintaining good health has been emphasized, the use of various nutrient supplements has drastically increased. Branched chain amino acids (BCAA), one group of indispensable amino acids, are known to be the main dietary factors that exert anabolic effects on proteins (Volpi et al. 2003). Among these, leucine increases the activation of mTOR, ribosomal protein S6 kinase (S6K1), and eukaryotic initiation factor 4E binding protein 1 (4E‐BP1) even at a single dose, and is directly involved in protein synthesis (Anthony et al. 2000). And Shamim et al. (2018) reported that muscle protein synthesis is closely related with satellite cell, leucine, and mTOR signaling pathway.

However, to identify the mechanism by which the intake of leucine, accompanied by regular exercise training, protein synthesis signaling, and the different stages of myogenesis, need to be elucidated. In adult muscles, primary myogenesis occurs upon the activation of satellite cells, located between the basal lamina and the plasma membrane (Mauro 1961). Satellite cells, muscle stem cells that exist within skeletal muscles, induce the expression of myogenic transcription factors (MRFs: MyoD, Myf5, myogenin, and MRF4) that are intrinsic to the quiescence, proliferation, differentiation, and self‐renewal stages of skeletal muscles. Satellite cells, that are normally quiescent, become activated by external stimuli, such as exercise and muscle damage, and initiate the myogenic program. Activated satellite cells gradually increase the expression of MyoD and myogenin that either repair damaged muscles, or induce the fusion of myoblasts with myotubes, or myoblast differentiation to form new myonuclei, based on the needs of skeletal muscle (Wang and Rudnicki 2011). Regenerated muscle fibers, containing newly fused satellite cells, display a central nucleus, with the nuclei of the muscle fibers located in the middle of the cytoplasm; these central nuclei gradually move to the periphery (Allen et al. 1999). Furthermore, embryonic myosin is a major marker for assessing skeletal muscle regeneration, due to satellite cell differentiation (Winter and Bornemann 1999). Averous et al. (2012) analyzed the effects of leucine treatment on muscle cells in vitro*,* by using C2C12 myoblasts. They observed that the expression of MyoD by satellite cells varied, depending on whether the cells were treated with/without leucine, and suggested that leucine may be involved in the differentiation of satellite cells and C2C12 myoblasts. On the other hand, Dai et al. (2015) used cell cultures of satellite cells, isolated from Sprague–Dawley rats, and reported increased levels of MyoD and myogenin expression, following leucine treatment. They also reported differences in the levels of satellite cell activation based on the leucine dose, and suggested that leucine may affect both the proliferation and differentiation of satellite cells. While the intake of leucine, which promotes protein synthesis, may be associated with myogenesis, the effects of leucine on myogenesis remain unclear. Moreover, previous studies on the effects of leucine intake at different doses combined with regular exercise training have only investigated the effects on protein synthesis signaling pathways, but only a few studies reported on muscle growth and satellite cell activity.

Therefore, we aim to investigate the effects of leucine intake and/or resistance exercise training for a period of 8 weeks on skeletal muscle growth and satellite cell activity, and compare their effects on the synthesis of proteins involved in muscle contraction for different doses of leucine.

## Materials and Methods

### Subjects

Ten‐week‐old male inbred Sprague–Dawley rats were used (Samtako, Gyeonggi, Korea) in this study. A total of 42 rats were randomly assigned to six groups (seven rats per group) and two rats were raised per cage with same group, where the conditions, temperature at 22 ± 2°C, humidity 50–60%, and a 12‐h light‐dark cycle. Fluid intake was not restricted. The rats ingested a diet consisting of 67.5% carbohydrates, 11.7% fat, and 20.8% proteins (Samtako, Gyeonggi, Korea). This study was approved by the Committee of Institutional Animal Care of the Korea National Sport University (KNSU‐IACUC‐2015‐07).

### Experimental design

The rats were assigned to the following six groups to investigate the effects of regular leucine intake and/or a 8‐week resistance training schedule on skeletal muscle growth and satellite cell activity: a control group with a normal diet and physical activity that was restricted to the inside of the cage (Con, *n* = 7), an exercise group subjected to a normal diet and resistance exercise training (Ex, *n* = 7), a 10% leucine group with an additional 10% dietary intake of leucine (Leu10, *n* = 7), a 50% leucine group with an additional 50% dietary intake of leucine (Leu50%, *n* = 7), a 10% leucine + exercise group with an additional 10% dietary intake of leucine after resistance exercise training (Leu10Ex, *n* = 7), and a 50% leucine + exercise group with an additional 50% dietary intake of leucine after resistance exercise training (Leu50Ex, *n* = 7) (Table 1). All leucine intake groups ingested leucine at the same time during the day. All exercise groups ingested leucine within 30 min after exercising.

**Table 1:** Experimental design

	Con	Leu10	Leu50	Ex	Leu10Ex	Leu50Ex
Exercise	‐	‐	‐	+	+	+
Leucine	‐	+10%	+50%	‐	+10%	+50%

Con: control group, Leu10: 10% leucine group, Leu50: 50% leucine group, Ex: exercise group, Leu10Ex: 10% leucine + exercise group, Leu50Ex: 50% leucine + exercise group.

### Exercise protocol

**Figure 1:**
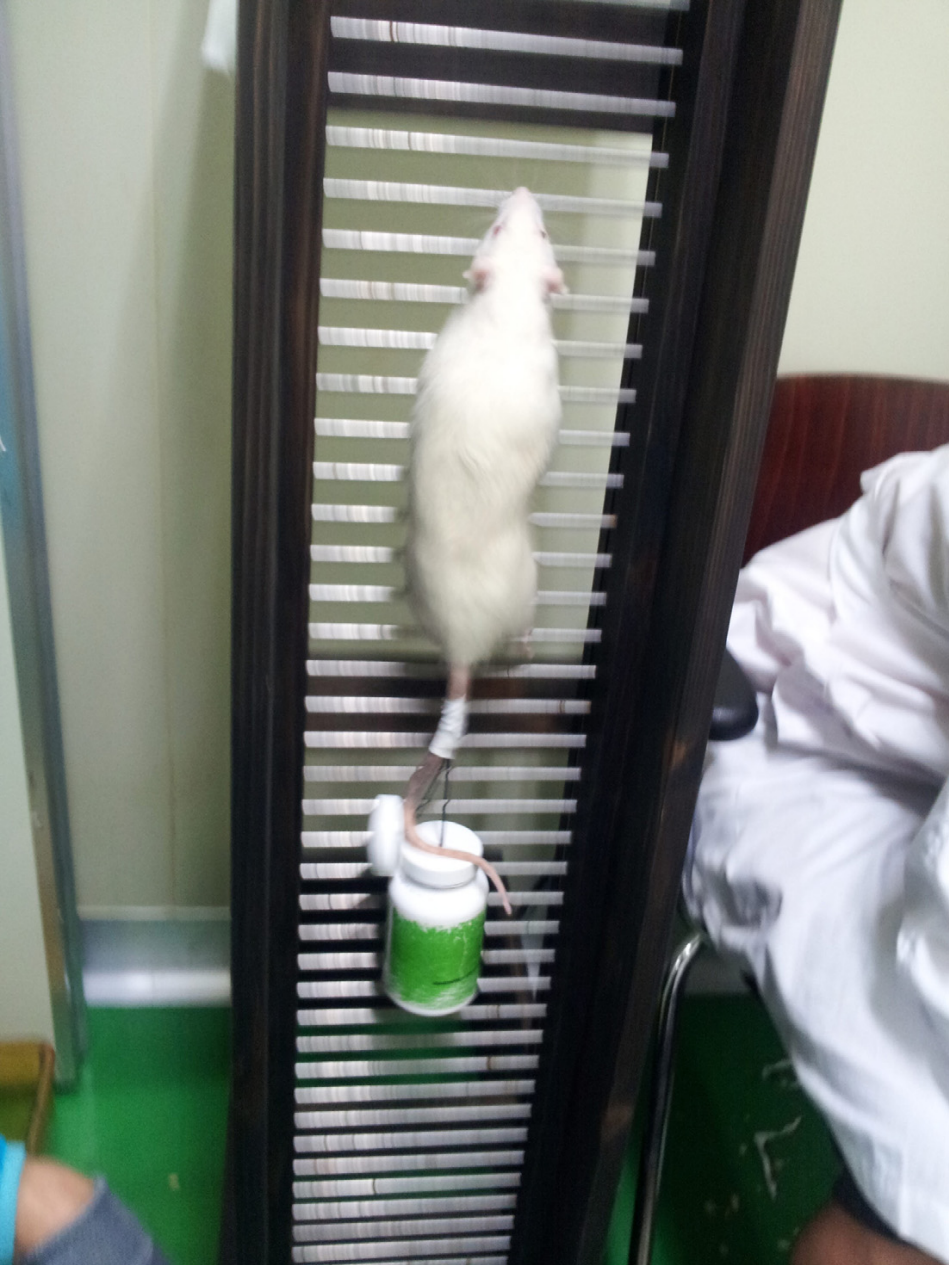
Climbing ladder exercise.

During resistance exercise training, the rats climbed a 1‐m‐long ladder with rungs that were 2 cm apart and inclined at 85°, three times per week, for 8 weeks (Fig. 1). Before performing the ladder climbing exercises, the rats performed habituation exercises without any load. Assuming the initial climb carrying load is the body weight of animal. The ladder climbing exercise was performed at 50%, 75%, 90%, and 100% of body weight, once each, and the load was increased by 30 g for each subsequent repetition up to 10 repetitions (Table 2). Training was terminated even if rats could not complete all ten repetitions due to the heavy load. The final load at the end of the training was set to 1 RM, and was used as the baseline intensity for the next training. The training load was increased by adding a coin with a fixed mass, or a load, onto the rat's tail. The rats had a 2‐minute break between each repetition (Hornberger and Farrar 2004).

**Table 2:** Climbing ladder daily exercise protocol

	1st	2nd	3rd	4th	5th	6th	7th	8th	9th	10th
Intensity	50% RM	75% RM	90% RM	100% RM	+30 g	+30 g	+30 g	+30 g	+30 g	+30 g

RM: repetition maximum.

### Leucine administration

Considering the amount of leucine that must be ingested by rats in 24 h (1.35 g/kg.wt) as the baseline, the 10% leucine group ingested an additional 0.135 g/kg.wt and the 50% leucine group ingested an additional 0.675 g/kg.wt of leucine (Crozier et al. 2005). Powdered leucine was dissolved in water and orally administered to the rats at the same time during the day using a Gavage needle. On the days of exercise training, leucine was administered within 30 min after exercise.

### Muscle sampling

Once the 8‐week‐long leucine ingestion and climbing exercises were over, the rats were sacrificed after they had fasted for approximately 12 h, 48 h after the last exercise, and 24 h after the last leucine administration. Several previous studies have reported that an additional protein intake affects fast‐twitch fibers, rather than slow‐twitch fibers (Hartman et al. 2007; Farup et al. 2014; Reidy et al. 2017). Accordingly, the rats were anesthetized using inhalation anesthetics (isoflurane), and the extensor digitorum longus (EDL) muscle, about 98% of which contains type II fibers, was removed (Armstrong and Phelps 1984). Some of the collected muscle tissues were pretreated with the optimal cutting temperature (O.C.T) compounds (Tissue‐Tek; Sakura Finetek Europe, California, U.S.A) for microscopy purposes, and the rest were flash frozen in liquid nitrogen and stored at −80°C for protein analysis.

### Satellite cells and myonuclei staining

To count the number of satellite cells and myonuclei, muscle cells were analyzed using immunohistochemistry, under a confocal microscope (Leica Microsystems, Wetzlar, Germany). The muscle tissues, pretreated with O.C.T compounds at −80°C, were cryo‐cut into 10‐㎛‐thick pieces, using a microtome (Leica, Wetzlar, Germany) at −20°C. The muscle tissues were placed on a slide glass, and fixed in 4% PFA for 5 min. To remove oil within the muscle tissues, these were submerged in precooled methanol at −20°C for 10 min, and blocking was performed using a 9:1 donkey serum and 0.1% triton/PBS blocking solution. To stain satellite cells, a mixture containing a primary anti‐Pax7 (MAB 1675, R&D system, USA) antibody and 1% BSA/TPBS solution at a 1:100 ratio was added to the muscle tissues, and their fasciae were stained using a primary anti‐Laminin (L9393, Sigma, USA) antibody. The muscle tissues were then incubated with primary antibodies overnight at 4°C. The next day, they were washed three times for 10 min using 0.1% triton/PBS. Next, the muscle tissues were incubated with secondary antibodies directed against the primary antibodies (Alexa fluor 594 donkey anti‐rabbit IgG antibody or Alexa fluor 488 donkey anti‐mouse IgG antibody), mixed with 1% BSA/TBPS at 1:500 at room temperature (20–25°C) for 4 h. After three washes with 0.1% triton/PBS for 10 min, the mounting medium (H‐1200, Vector Laboratories, California, U.S.A) containing 4,6‐diamidino‐2‐phenylindole (DAPI) was added to the muscle tissues for myonuclear staining, and the muscle tissues were fixed with a cover glass. Over 250 muscle fibers were analyzed per sample.

### Myofiber remodeling

Skeletal muscle regeneration can be quantified based on the ratio of the total number of myofibers to the number of embryonic myosin fibers. Myofiber regeneration was quantified by immunohistochemistry. Muscle tissues were fixed in 4% PFA, and treated with an antiembryonic myosin antibody (F1.652, Developmental Studies Hybridoma Bank, USA) and an anti‐Laminin (L9393, Sigma, USA) antibody, diluted in 1% BSA/TPBS at 1/100, to distinguish the fibers. Following an overnight incubation, they were treated with secondary antibodies against the primary antibodies (Alexa fluor 594 donkey anti‐rabbit IgG antibody, anti‐mnryonic or Alexa fluor 488 donkey anti‐mouse IgG antibody, anti‐Laminin). Muscle images were obtained using confocal microscopy.

### Western blot analysis

The muscle tissues stored at −80°C were ground in liquid nitrogen. After shaking in lysis buffer (25 mmol/L Tris‐Cl pH 7.5, 250 mmol/L NaCl, 5 mmol/L EDTA, 1% NP‐40, 1 mmol/L phenylmethylsulfonyl fluoride, and 5 mmol/L dithiothreitol) at 4°C, the tissues were incubated overnight. The next day, the tissues were centrifuged at 20,000*g* at 4°C, and the supernatant was retained. The protein levels in the supernatant were quantified, and 2x SDS loading buffer (60 mmol/L Tris pH 6.8, 25% glycerol, 2% SDS, 14.4 mmol/L 2‐mercaptoethanol, and 0.1% Bromophenol Blue) was added to the samples. Samples and standard markers (SDS‐PAGE Molecular Weight Standard, BioRad, USA) were loaded on a 12% gel (30% acrylamide, 1.5 mol/L Tris pH 8.8, 10% Ammonium persulfate, TEMED) and a 5% stacking gel (30% acrylamide, 1 mol/L Tris pH 6.8, 10% Ammonium persulfate, TEMED), and electrophoresis was performed.

To transfer proteins from the gels onto membranes, a nitrocellulose blotting membrane (Life Sciences, Germany) and 3M paper (Whatman) were sequentially placed onto a Mini transblot cell (BioRad, USA), and the transfer was performed at 80 V for 1.5 h in transfer buffer (190 mmol/L glycine, 50 mmol/L Tris‐base, 0.05% SDS, 20% methanol). After the protein transfer was complete, the membranes were subjected to blocking in 5% bovine serum albumin for 1.5 h. Then, 10 *μ*L of anti‐MyoD (sc‐71629, Santa Cruz, USA), antimyogenin (sc‐12732, Santa Cruz, USA), and anti‐IGF‐1 (sc‐9013, Santa Cruz, USA) antibodies were added to each membrane, and incubated overnight at 4°C. After three washes with TBST for 10 min, the membranes were incubated with secondary antibodies against the primary antibodies, diluted in 4% skim milk at a 1:5000 dilution, at room temperature, for 1.5 h. Once the incubation was complete, the membranes were washed again with TBST, three times for 10 min. Proteins were visualized using the WBLR solution (Luminata Crescendo Western HRP Substrate, Millipore, USA), and quantified by scanning using the ChemiDoc XRS system (Quantity One 1‐D Analysis Software, Bio‐Rad, USA).

### Statistics

All obtained results were expressed as the mean ± standard deviation, by using the SPSS/PASW statistics software, version 18. A one‐way ANOVA was used to find significant differences among groups (Con, Leu10, Leu50, Ex, Leu10Ex, and Leu50Ex). The Tukey HSD test was used for the post hoc analysis. The level of statistical significance was set at *α *= 0.05.

## Results

### Body weight and maximal carrying load

The largest weight gain of 138.7 g (139.9%) was observed in the Leu50Ex group on the 8th week relative to the 1st week; however, no significant differences in weight gain were observed among the groups (Table 3). The Leu50Ex group had the highest maximal carrying load of 1231.2 ± 161.3 g on the 8th week among all exercise groups. However, the Leu10Ex group had the largest increase in the maximal carrying load, at 847.4 ± 1.9 g (338.9%) after 8 weeks of training, relative to the 1st week, but no significant difference among the groups (Table 4).

**Table 3:** Body weight

	1 week	2 weeks	3 weeks	4 weeks	5 weeks	6 weeks	7 weeks	8 weeks
Con	354.6 ± 8.9	375.4 ± 21	391.7 ± 50.7	420.1 ± 49.3	468.7 ± 51.3	482.4 ± 56.3	493.9 ± 55.7	474.3 ± 55.7
Leu10	340.7 ± 6	378.5 ± 12.7	409.3 ± 23.2	401.4 ± 28.6	455.3 ± 37.8	463.4 ± 48.7	475 ± 44.5	461.7 ± 38.5
Leu50	346.9 ± 6.4	380.9 ± 35.8	405.2 ± 21.7	431.7 ± 39.2	458.1 ± 27	476.6 ± 27.3	480 ± 26.1	469.6 ± 25.1
Ex	348.9 ± 6.3	376.4 ± 8.1	407.7 ± 12.9	415.7 ± 25.6	460.4 ± 16.2	473.3 ± 15.1	481.9 ± 20.1	457.4 ± 16.4
Leu10Ex	351.3 ± 8.9	375.6 ± 8.8	401.6 ± 12.4	405.1 ± 13.5	437.4 ± 20.6	459.7 ± 17.7	460.3 ± 20	443.6 ± 18.1
Leu50Ex	347.7 ± 9.3	391.5 ± 16.9	425 ± 31.4	427.2 ± 26.3	490.5 ± 30.3	492.8 ± 37.4	498.3 ± 39	486.3 ± 35.4

Unit: g, Con: control group, Leu10: 10% leucine group, Leu50: 50% leucine group, Ex: exercise group, Leu10Ex: 10% leucine + exercise group, Leu50Ex: 50% leucine + exercise group.

**Table 4:** Maximal carrying load

	1 week	2 weeks	3 weeks	4 weeks	5 weeks	6 weeks	7 weeks	8 weeks
Ex	435.3 ± 79	469.9 ± 48.6	500.6 ± 45.6	575.6 ± 148.4	803.1 ± 71.2	900.4 ± 105.7	1000.7 ± 120.4	1167.4 ± 161.9
Leu10Ex	354.7 ± 101.6	454.7 ± 68.4	507.3 ± 57.1	641.9 ± 112.7	820.7 ± 111.5	961.9 ± 115.1	1092.4 ± 124.4	1202.1 ± 162.8
Leu50Ex	400.7 ± 94.2	514.5 ± 92.5	500.2 ± 57.1	548.2 ± 61.3	857.2 ± 145.2	937.5 ± 125.6	1096.2 ± 130.9	1231.2 ± 161.3

Unit: g, Con: control group, Leu10: 10% leucine group, Leu50: 50% leucine group, Ex: exercise group, Leu10Ex: 10% leucine + exercise group, Leu50Ex: 50% leucine + exercise group.

### Muscle fiber area

**Figure 2:**
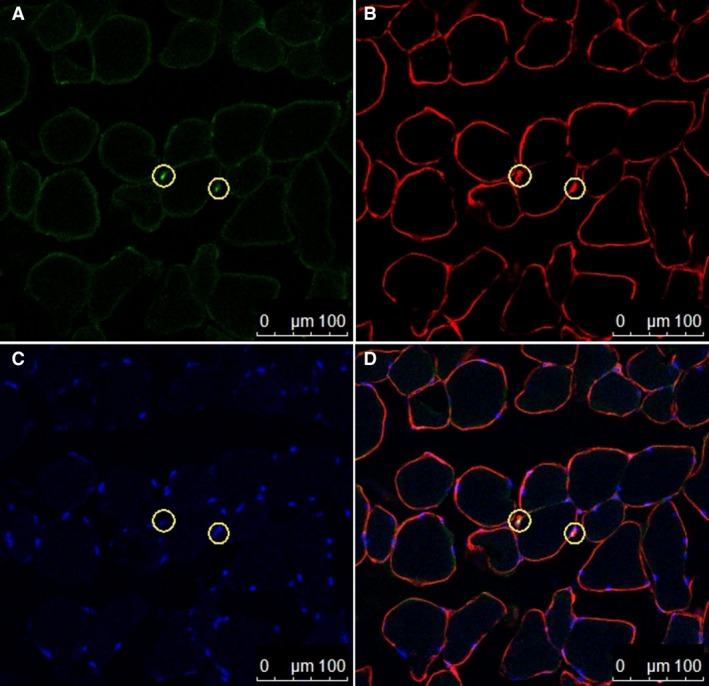
Immunohistochemical detection of satellite cells (Pax7, green). (A), laminin (antilaminin, red), (B), nuclei (DAPI, blue), and (C) merged. The two yellow circles represent satellite cells for which the green and blue signals exactly overlap. Scale bar = 100 *μ*m.

While no differences in the single fiber cross‐sectional area were found among the Con (1879.7 ± 175.8 *μ*m^2^), Leu10 (2387.8 ± 824.3 *μ*m^2^, 127%), and Leu50 (2208 ± 698.6 *μ*m^2^, 117%) groups (*P* > 0.05), the Ex (2655.7 ± 258 *μ*m^2^, 141.3%), Leu10Ex (2695.1 ± 213.4 *μ*m^2^, 143.4%), and Leu50Ex (2703 ± 155.5 *μ*m^2^, 143.8%) groups displayed increased cross‐sectional areas relative to those in the Con group (*P* < 0.05). However, no significant differences in the cross‐sectional area were found among the Ex, Leu10Ex, and Leu50Ex groups (*P* > 0.05) (Fig. 2).

### Satellite cells

**Figure 3:**
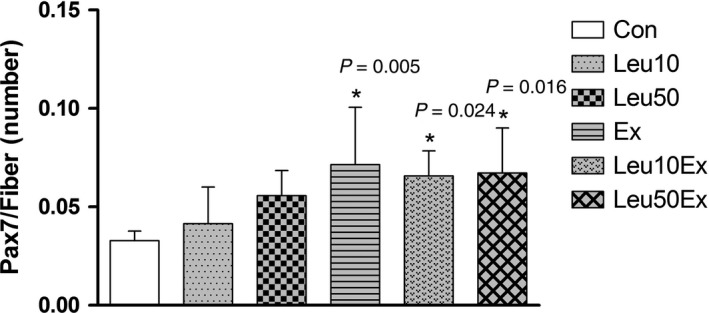
Satellite cell (Pax7/Fiber) numbers in EDL muscles. Con: control group, Leu10: 10% leucine group, Leu50: 50% leucine group, Ex: exercise group, Leu10Ex: 10% leucine + exercise group, Leu50Ex: 50% leucine + exercise group. * *P *<* *0.05, significant difference from Con.

No significant differences in the number of satellite cells were found for the Leu10 (0.04 ± 0.01, 133%) and Leu50 (0.05 ± 0.01, 166%) groups relative to the Con group (0.03 ± 0.004) (*P* > 0.05). However, this number was significantly increased in the Ex (0.07 ± 0.03, 233%), Leu10Ex (0.07 ± 0.01, 233%), and Leu50Ex (0.07 ± 0.02, 233%) groups relative to that in the Con group (*P* < 0.05); no significant differences were observed among these three groups (*P* > 0.05) (Figs. 2 and 3).

### Myonuclei and central nuclei

**Figure 4:**
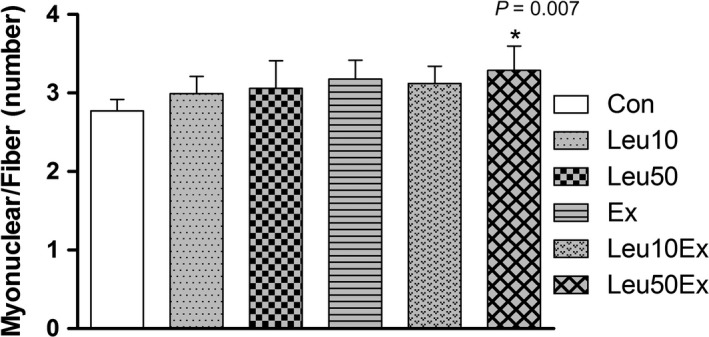
Myonuclear number (myonuclear/fiber) in EDL muscles. Con: control group, Leu10: 10% leucine group, Leu50: 50% leucine group, Ex: exercise group, Leu10Ex: 10% leucine + exercise group, Leu50Ex: 50% leucine + exercise group. * *P *<* *0.05, significant difference from Con.

**Figure 5:**
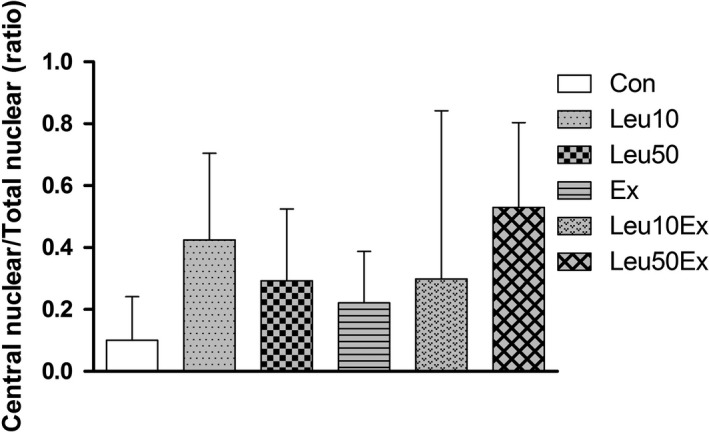
Ratio of central nuclei in EDL muscles. Con: control group, Leu10: 10% leucine group, Leu50: 50% leucine group, Ex: exercise group, Leu10Ex: 10% leucine + exercise group, Leu50Ex: 50% leucine + exercise group.

**Figure 6:**
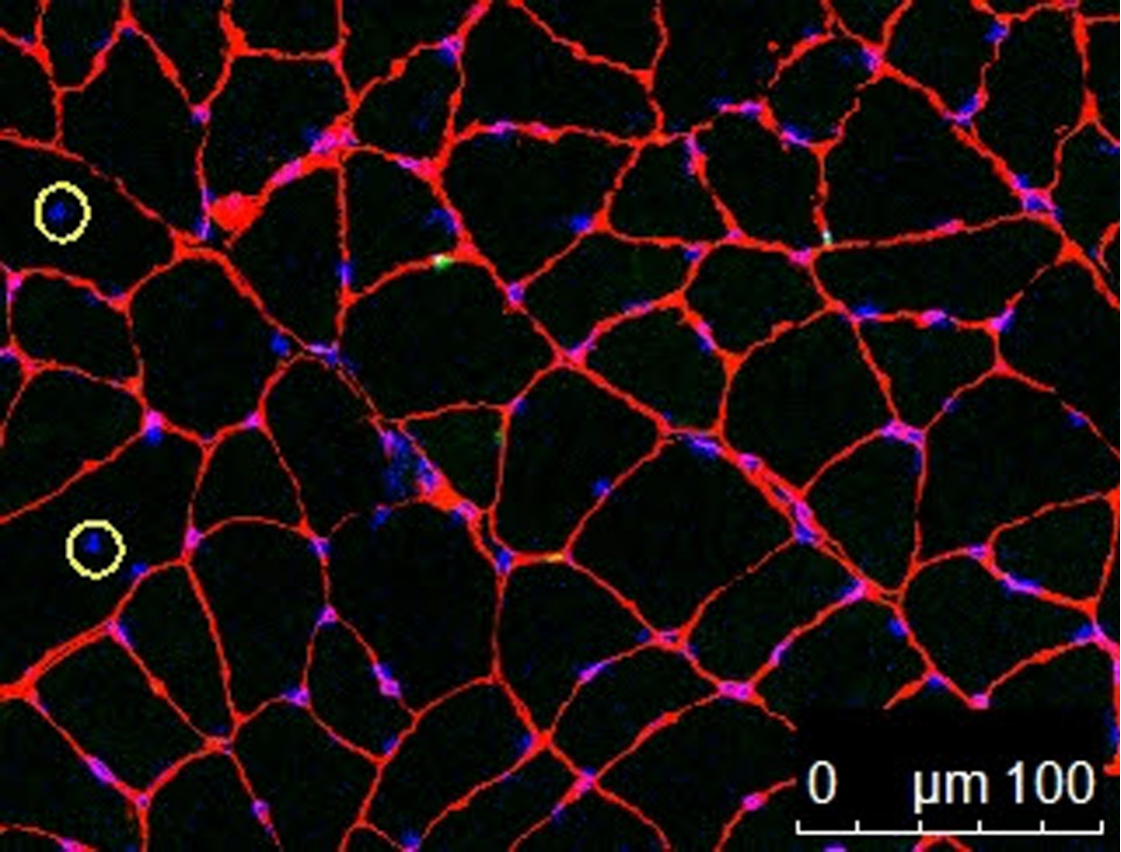
Immunohistochemical detection of central nuclei. The two yellow circles represent central nuclei. Scale bar = 100 *μ*m.

The myonuclear number (myonuclear/fiber) increased in the Leu50Ex (3.29 ± 0.3, 118.7%) group relative to that in the Con (2.77 ± 0.1) group (*P* < 0.05). No significant differences in the myonuclear number relative to the Con group were found for the Leu10 (2.99 ± 0.2, 107%), Leu50 (3.06 ± 0.4, 110%), Ex (3.18 ± 0.2, 114%), and Leu10Ex (3.12 ± 0.2, 112%) groups (*P* > 0.05) (Fig. 4). The central nuclear ratio was observed to increase due to exercise or leucine treatment, relative to the Con group; however, the ratio largely varied among the samples (*P* > 0.05) (Figs. 5 and 6).

### Myofiber remodeling

**Figure 7:**
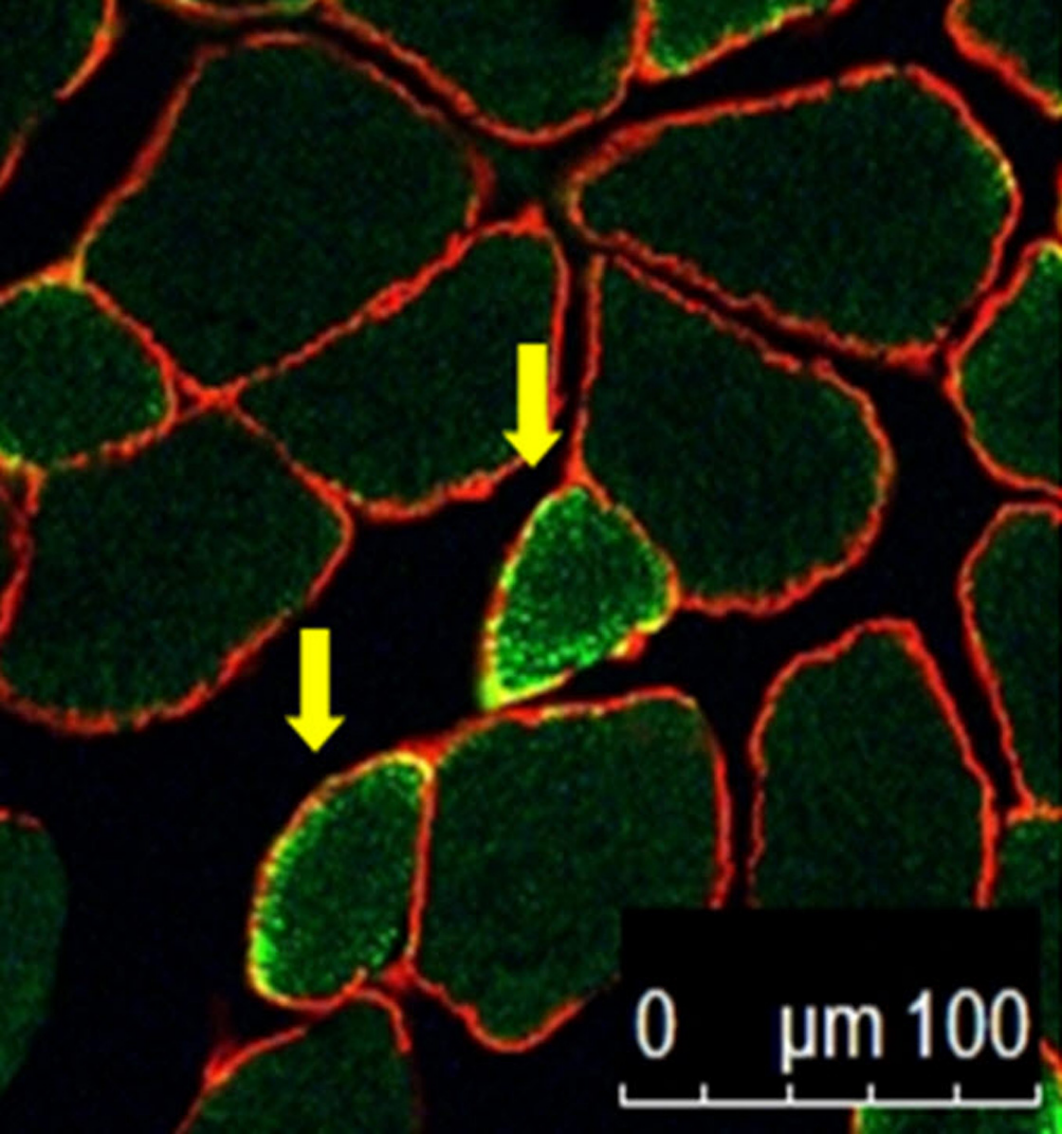
Immunohistochemical detection of the heavy chain of embryonic myosin. The two yellow arrows indicate embryonic myosin‐positive fibers. Scale bar = 100 *μ*m.

**Figure 8:**
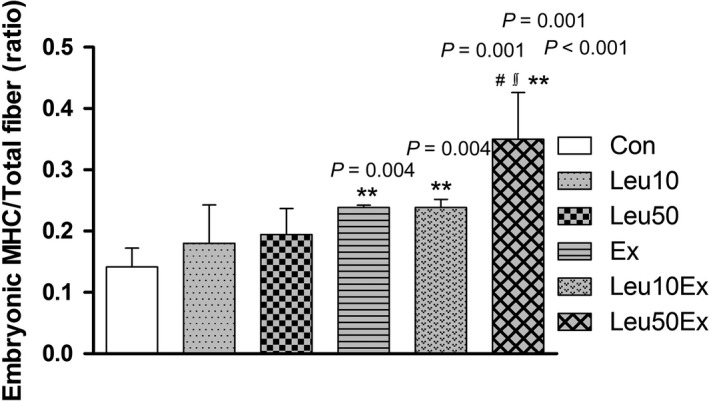
Embryonic MHC in EDL muscles. MHC: myosin heavy chain, Con: control group, Leu10: 10% leucine group, Leu50: 50% leucine group, Ex: exercise group, Leu10Ex: 10% leucine + exercise group, Leu50Ex: 50% leucine + exercise group. ** *P *<* *0.005, significant difference from Con, # *P *<* *0.005, significant difference from Ex, ∬ *P *<* *0.005, significant difference from Leu10Ex.

While no differences in the embryonic myosin heavy chain/total fiber ratio relative to the Con (0.14 ± 0.03) group were found for the Leu10 (0.18 ± 0.06, 128%) and Leu50 (0.19 ± 0.04, 135%) groups, the ratio increased in the Ex (0.23 ± 0.003, 164.3%), Leu10Ex (0.24 ± 0.01, 171.4%), and Leu50Ex (0.35 ± 0.08, 250%) groups relative to the Con group (*P* < 0.005). Of these, the Leu50Ex (152.1%, *P* = 0.001, and 145.8%, *P* = 0.001, for Ex and Leu10Ex, respectively) group exhibited a higher number of embryonic myosin‐positive fibers than the Ex and Leu10Ex groups (Figs. 7 and 8).

### MyoD and myogenin protein expression

**Figure 9:**
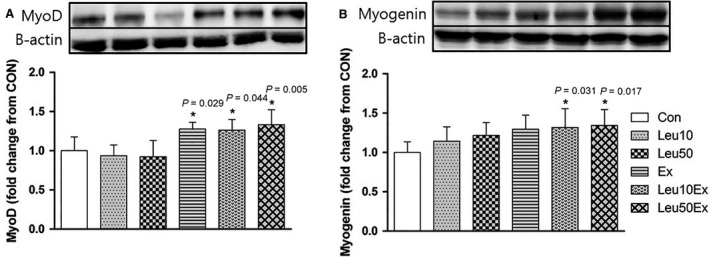
MyoD (A) and myogenin (B) protein expression in EDL muscles. Con: control group, Leu10: 10% leucine group, Leu50: 50% leucine group, Ex: exercise group, Leu10Ex: 10% leucine + exercise group, Leu50Ex: 50% leucine + exercise group. * *P *<* *0.05, significant difference from Con.

No significant differences in the level of MyoD protein expression relative to that of the Con group were found for the Leu10 (93.7%, *P* = 0.978) and Leu50 (92.3%, *P* = 0.947) groups. However, Ex (127.7%, *P* = 0.029), Leu10Ex (126.2%, *P* = 0.044), and Leu50Ex (133.3%, *P* = 0.005) presented higher levels of protein expression than that of the Con group. No significant differences in the level of protein expression were found among these three exercise groups (*P* > 0.05) (Fig. 9A). No significant differences in the level of myogenin protein expression relative to that of the Con group were found for Leu10 (114.4%, *P* = 0.695) and Leu50 (121.8%, *P* = 0.262), while significant differences were found for the Leu10Ex (131.8%, *P* = 0.031) and Leu50Ex (134.3%, *P* = 0.017) groups relative to that of the Con group (Fig. 9B), and Ex group (130%, *P* = 0.054) tended increase.

### IGF‐1 protein expression

**Figure 10:**
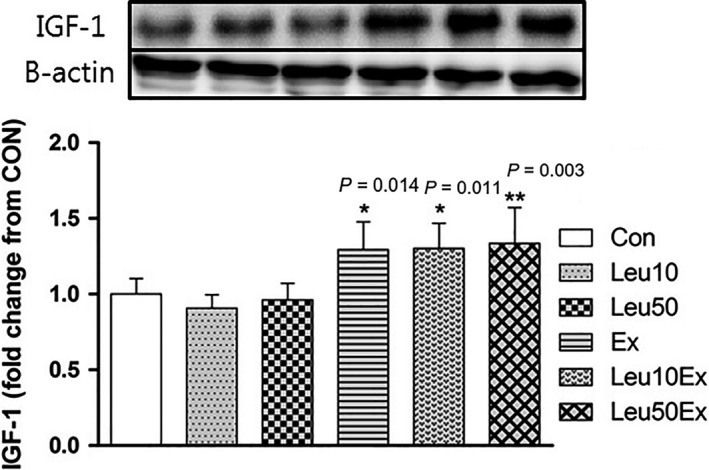
IGF‐1 protein expression in EDL muscles. Con: control group, Leu10: 10% leucine group, Leu50: 50% leucine group, Ex: exercise group, Leu10Ex: 10% leucine + exercise group, Leu50Ex: 50% leucine + exercise group. * *P *<* *0.05, significant difference from Con, ** *P* < 0.005, significant difference from Con.

No significant differences in the levels of Insulin‐like growth factor 1 (IGF‐1) protein expression relative to those in the Con group were found for the Leu10 (90.7%, *P* = 0.876) and Leu50 (96.2%, *P* = 0.998) groups; however, significant differences relative to the Con group were observed for the Ex (129.3%, *P* = 0.014), Leu10Ex (130.1%, *P* = 0.011), and Leu50Ex (133.6%, *P* = 0.003) groups. No significant differences were found among the three exercise groups (*P* > 0.05) (Fig. 10).

## Discussion

The major finding of this study is that an 8 weeks of regular leucine administration combined with exercise training can increase the number of myonuclei and embryonic myosin fibers by increasing satellite cell differentiation, while leucine administration alone did not induce to increase satellite cell differentiation and skeletal muscle hypertrophy regardless of the dose.

**Figure 11:**
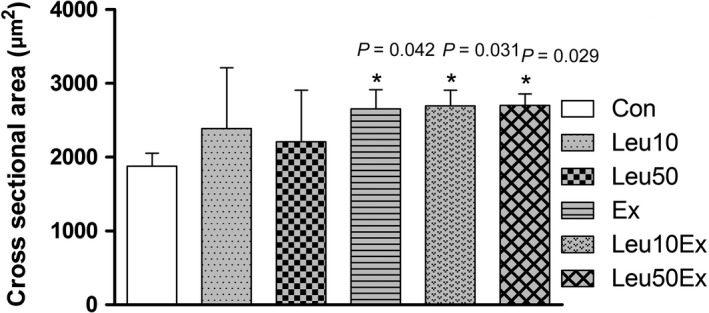
Cross‐sectional areas of EDL muscles. Con: control group, Leu10: 10% leucine group, Leu50: 50% leucine group, Ex: exercise group, Leu10Ex: 10% leucine + exercise group, Leu50Ex: 50% leucine + exercise group. * *P *<* *0.05, significant difference from Con.

Physical activity promotes both protein synthesis and degradation in skeletal muscles. Physical activity and an adequate nutrient intake effectively induce muscle growth (Phillips et al. 1997). The dietary intake of essential amino acids represents a major cause of the increase in the level of proteins synthesis involved in muscle contraction. Among BCAAs, leucine has the most direct effects on protein synthesis in skeletal muscles (Anthony et al. 2000). Leucine has been reported to increase protein synthesis by increasing the phosphorylation of proteins involved in the mTOR signaling pathway (Drummond et al. 2009). However, this study demonstrated that no significant differences in the myofiber cross‐sectional areas relative to those in the Con group were found for the Leu10% and Leu50% groups, comprising an additional 10% and 50% leucine intake to the ordinary diet, respectively. Conversely, the cross‐sectional areas of myofibers increased in the Ex (*P *=* *0.042), Leu10Ex (*P *=* *0.031), and Leu50Ex (*P *=* *0.029) groups that performed resistance training with or without leucine ingestion, relative to that of the Con group (Fig. 11). This finding suggests that the long‐term administration of leucine in the absence of resistance exercise training may not induce skeletal muscle hypertrophy. Previous studies claiming that leucine can induce hypertrophy by increasing the phosphorylation of proteins in protein synthesis signaling pathways, including the S6K1 and 4EBP1 signaling pathways, have mostly investigated the acute effects of leucine (Norton and Layman 2006; Dreyer et al. 2008; Reidy et al. 2017). In addition, Koopman et al. (2008), and Pereira et al. (2014) reported that leucine supplementation is effective during the early stages of protein synthesis, and induces skeletal muscle repair or regeneration, rather than hypertrophy. Perry et al. (2016) reported that while leucine ingestion combined with exercise may promote protein synthesis, the effects of leucine on protein synthesis are minimal because the signaling pathways involved in hypertrophy are mainly affected by the upregulation of IGF‐1 proteins following exercise. Our results are consistent with this finding. Hypertrophy was only observed in the Ex, Leu10Ex, and Leu50Ex groups that had higher levels of IGF‐1 expression relative to the Con group due to regular resistance exercise training (Fig. 11 and 10). Leucine and IGF‐1 act as catalysts that increase the activation of the mTOR signaling pathway in skeletal muscles, although through different mechanisms. Our previous finding of 8‐week leucine supplementation in combined with exercise also support this notion that exercise with leucine supplementation can induce higher p70S6K protein phosphorylation than leucine supplementation only, even though in flexor hallusis longus (FHL)(Gil and Kim 2015). However, Han et al. (2008) reported that the simultaneous stimulation of the two signaling mechanisms does not result in a synergistic effect on protein synthesis. Our findings regarding the differences in the cross‐sectional areas among the Ex, Leu10Ex, and Leu50Ex groups are consistent with the findings of Han et al. Therefore, the skeletal muscle hypertrophy observed following leucine ingestion in combination with exercise may be mainly due to the exercise training, which induces muscle growth by upregulating IGF‐1, rather than leucine.

Satellite cells undergo activation, proliferation, and differentiation, and eventually form new myonuclei or fuse into myofibers as myotubes to induce myogenesis. The metabolism of satellite cells is essential in controlling skeletal muscle hypertrophy and improving muscle quality (Blaauw and Reggiani 2014). A recent in vitro experiment suggested that leucine administration increases the expression of MyoD and myogenin by satellite cells, thereby activating satellite cells (Shamim et al. 2018). However, while an 8 weeks of regular leucine administration did not affect the number of satellite cells, this number significantly increased in the Ex group that was subjected to exercise training without leucine ingestion in this study (Fig. 3). Previous studies have reported that leucine ingestion downregulates rapamycin, which inhibits the mTOR signaling pathway, thereby activating satellite cells, and accelerating the myogenic process (Erbay and Chen 2001; Shu and Houghton 2009). However, contrary to the findings of these studies, the continuous administration of leucine for 8 weeks in the absence of exercise did not affect satellite cell differentiation and muscle hypertrophy in this study. Our findings suggest that a stimulus initiated by exercise has a major effect on the proliferation and differentiation of satellite cells that is confirmed by our cross‐sectional area results. Previous studies that have reported on the effects of a continuous dietary intake on protein synthesis have mostly used whey proteins (Anthony et al. 2000), essential amino acids, or complex amino acids such as total BCAAs (Shamim et al. 2018) rather than leucine alone. Therefore, even if leucine ingestion affects the very early phases of protein synthesis and/or protein signaling pathways involved in myogenic activation in muscles, additional research on the physiological effects of a continuous leucine intake is necessary.

The number of myonuclei (Fig. 4) and embryonic myosin fibers (Fig. 8) in skeletal muscles significantly increased in the Leu50Ex group, subjected to an 8‐week course of high‐dose leucine administration in combination with resistance exercise training, relative to those of the Ex and Leu10Ex groups. The expression of myogenin, which is upregulated during the last stages of satellite cell differentiation, was also significantly increased in the Leu10Ex and Leu50Ex groups, which performed resistance exercise in addition to leucine ingestion (Fig. 9B). Kadi et al. (1998) reported increased numbers of embryonic MHC fibers and central nuclei owing to the active muscle regeneration induced by adequate levels of physical activity in patients with myalgia. The increased levels of embryonic MHC indicate the active regeneration and remodeling of skeletal muscles. The continuous administration of high doses of leucine in combination with resistance exercise training may improve the muscle growth, by inducing the differentiation of satellite cells into myonuclei and embryonic myosin fibers. Cribb et al. (2007) reported that while protein intake may not affect the cross‐sectional areas of myofibers, it increases muscle strength. The present study result has similar tendency to this previous study. However, the changes in the maximal carrying load were not significantly greater among the groups (Table 4). Therefore, leucine administration in combination with resistance exercise training may improve muscle metabolism by leading to the formation of new myonuclei and embryonic myosin fibers, rather than by inducing hypertrophy, with additional effects from regular exercise.

In summary, an 8‐week leucine administration alone did not increase satellite cell differentiation and skeletal muscle hypertrophy up to 150% of normal daily leucine intake. However, when combined with exercise training, it leads to increase in the differentiation of satellite cells, and increases the cross‐sectional areas of myofibers. Therefore, a continuous leucine intake in combination with regular exercise may improve anabolic effects in skeletal muscles and muscle quality by activating satellite cell differentiation more effectively than leucine ingestion alone.

## Conflict of Interest

The authors declare that have no conflict of interest.

## References

[phy213725-bib-0001] Allen, D. L. , R. R. Roy , and V. R. Edgerton . 1999 Myonuclear domains in muscle adaptation and disease. Muscle Nerve 22:1350–1360. 10487900 10.1002/(sici)1097-4598(199910)22:10<1350::aid-mus3>3.0.co;2-8

[phy213725-bib-0002] Anthony, J. C. , F. Yoshizawa , T. G. Anthony , T. C. Vary , L. S. Jefferson , and S. R. Kimball . 2000 Leucine stimulates translation initiation in skeletal muscle of postabsorptive rats via a rapamycin‐sensitive pathway. J. Nutr. 130:2413–2419. 11015466 10.1093/jn/130.10.2413

[phy213725-bib-0003] Armstrong, R. B. , and R. O. Phelps . 1984 Muscle fiber type composition of the rat hindlimb. Am. J. Anat. 171:259–272. 6517030 10.1002/aja.1001710303

[phy213725-bib-0004] Averous, J. , J. C. Gabillard , I. Seiliez , and D. Dardevet . 2012 Leucine limitation regulates myf5 and myoD expression and inhibits myoblast differentiation. Exp. Cell Res. 318:217–227. 22079119 10.1016/j.yexcr.2011.10.015

[phy213725-bib-0005] Blaauw, B. , and C. Reggiani . 2014 The role of satellite cells in muscle hypertrophy. J. Muscle Res. Cell Motil. 35:3–10. 24505026 10.1007/s10974-014-9376-y

[phy213725-bib-0006] Clynes, M. A. , M. H. Edwards , B. Buehring , E. M. Dennison , N. Binkley , and C. Cooper . 2015 Definitions of sarcopenia: associations with previous falls and fracture in a population sample. Calcif. Tissue Int. 97:445–452. 26223791 10.1007/s00223-015-0044-z PMC4601152

[phy213725-bib-0007] Cribb, P. J. , A. D. Williams , C. G. Stathis , M. F. Carey , and A. Hayes . 2007 Effects of whey isolate, creatine, and resistance training on muscle hypertrophy. Med. Sci. Sports Exerc. 39:298–307. 17277594 10.1249/01.mss.0000247002.32589.ef

[phy213725-bib-0008] Crozier, S. J. , S. R. Kimball , S. W. Emmert , J. C. Anthony , and L. S. Jefferson . 2005 Oral leucine administration stimulates protein synthesis in rat skeletal muscle. J. Nutr. 135:376–382. 15735066 10.1093/jn/135.3.376

[phy213725-bib-0009] Dai, J. M. , M. X. Yu , Z. Y. Shen , C. Y. Guo , S. Q. Zhuang , and X. S. Qiu . 2015 Leucine promotes proliferation and differentiation of primary preterm rat satellite cells in part through mTORC1 signaling pathway. Nutrients 7:3387–3400. 26007333 10.3390/nu7053387 PMC4446757

[phy213725-bib-0010] Dreyer, H. C. , S. Fujita , J. G. Cadenas , D. L. Chinkes , E. Volpi , and B. B. Rasmussen . 2006 Resistance exercise increases AMPK activity and reduces 4E‐BP1 phosphorylation and protein synthesis in human skeletal muscle. J. Physiol. 576:613–624. 16873412 10.1113/jphysiol.2006.113175 PMC1890364

[phy213725-bib-0011] Dreyer, H. C. , M. J. Drummond , B. Pennings , S. Fujita , E. L. Glynn , D. L. Chinkes , et al. 2008 Leucine‐enriched essential amino acid and carbohydrate ingestion following resistance exercise enhances mTOR signaling and protein synthesis in human muscle. Am. J. Physiol. Endocrinol. Metab. 294:E392–E400. 18056791 10.1152/ajpendo.00582.2007 PMC2706121

[phy213725-bib-0012] Drummond, M. J. , H. C. Dreyer , C. S. Fry , E. L. Glynn , and B. B. Rasmussen . 2009 Nutritional and contractile regulation of human skeletal muscle protein synthesis and mTORC1 signaling. J. Appl. Physiol. (1985) 106:1374–1384. 19150856 10.1152/japplphysiol.91397.2008 PMC2698645

[phy213725-bib-0013] Erbay, E. , and J. Chen . 2001 The mammalian target of rapamycin regulates C2C12 myogenesis via a kinase‐independent mechanism. J. Biol. Chem. 276:36079–36082. 11500483 10.1074/jbc.C100406200

[phy213725-bib-0014] Farup, J. , S. K. Rahbek , S. Riis , M. H. Vendelbo , F. Paoli , and K. Vissing . 2014 Influence of exercise contraction mode and protein supplementation on human skeletal muscle satellite cell content and muscle fiber growth. J. Appl. Physiol. (1985) 117:898–909. 25103976 10.1152/japplphysiol.00261.2014 PMC4280155

[phy213725-bib-0015] Gil, J. H. , and C. K. Kim . 2015 Effects of different doses of leucine ingestion following eight weeks of resistance exercise on protein synthesis and hypertrophy of skeletal muscle in rats. J. Exerc. Nutrition Biochem. 19:31–38. 10.5717/jenb.2015.19.1.31 PMC4424444 25960953

[phy213725-bib-0016] Han, B. , J. Tong , M. J. Zhu , C. Ma , and M. Du . 2008 Insulin‐like growth factor‐1 (IGF‐1) and leucine activate pig myogenic satellite cells through mammalian target of rapamycin (mTOR) pathway. Mol. Reprod. Dev. 75:810–817. 18033679 10.1002/mrd.20832

[phy213725-bib-0017] Hartman, J. W. , J. E. Tang , S. B. Wilkinson , M. A. Tarnopolsky , R. L. Lawrence , A. V. Fullerton , et al. 2007 Consumption of fat‐free fluid milk after resistance exercise promotes greater lean mass accretion than does consumption of soy or carbohydrate in young, novice, male weightlifters. Am. J. Clin. Nutr. 86:373–381. 17684208 10.1093/ajcn/86.2.373

[phy213725-bib-0018] Jr Hornberger, T. A. , and R. P. Farrar . 2004 Physiological hypertrophy of the FHL muscle following 8 weeks of progressive resistance exercise in the rat. Can. J. Appl. Physiol. 29:16–31. 15001801 10.1139/h04-002

[phy213725-bib-0019] Kadi, F. , and L. E. Thornell . 2000 Concomitant increases in myonuclear and satellite cell content in female trapezius muscle following strength training. Histochem. Cell Biol. 113:99–103. 10766262 10.1007/s004180050012

[phy213725-bib-0020] Kadi, F. , G. Hagg , R. Hakansson , S. Holmner , G. S. Butler‐Browne , and L. E. Thornell . 1998 Structural changes in male trapezius muscle with work‐related myalgia. Acta Neuropathol. 95:352–360. 9560012 10.1007/s004010050810

[phy213725-bib-0021] Koopman, R. , L. B. Verdijk , M. Beelen , M. Gorselink , A. N. Kruseman , A. J. Wagenmakers , et al. 2008 Co‐ingestion of leucine with protein does not further augment post‐exercise muscle protein synthesis rates in elderly men. Br. J. Nutr. 99:571–580. 17697406 10.1017/S0007114507812013

[phy213725-bib-0022] Kosek, D. J. , J. S. Kim , J. K. Petrella , J. M. Cross , and M. M. Bamman . 2006 Efficacy of 3 days/wk resistance training on myofiber hypertrophy and myogenic mechanisms in young vs. older adults. J. Appl. Physiol. (1985) 101:531–544. 16614355 10.1152/japplphysiol.01474.2005

[phy213725-bib-0023] Mauro, A. 1961 Satellite cell of skeletal muscle fibers. J. Biophys. Biochem. Cytol. 9:493–495. 13768451 10.1083/jcb.9.2.493 PMC2225012

[phy213725-bib-0024] Norton, L. E. , and D. K. Layman . 2006 Leucine regulates translation initiation of protein synthesis in skeletal muscle after exercise. J. Nutr. 136:533S–537S. 16424142 10.1093/jn/136.2.533S

[phy213725-bib-0025] Pereira, M. G. , I. L. Baptista , E. O. Carlassara , A. S. Moriscot , M. S. Aoki , and E. H. Miyabara . 2014 Leucine supplementation improves skeletal muscle regeneration after cryolesion in rats. PLoS ONE 9:e85283. 24416379 10.1371/journal.pone.0085283 PMC3885703

[phy213725-bib-0026] Perry, R. A. Jr , L. A. Brown , D. E. Lee , J. L. Brown , J. I. Baum , N. P. Greene , et al. 2016 Differential effects of leucine supplementation in young and aged mice at the onset of skeletal muscle regeneration. Mech. Ageing Dev. 157:7–16. 27327351 10.1016/j.mad.2016.05.007 PMC5002371

[phy213725-bib-0027] Phillips, S. M. , K. D. Tipton , A. Aarsland , S. E. Wolf , and R. R. Wolfe . 1997 Mixed muscle protein synthesis and breakdown after resistance exercise in humans. Am. J. Physiol. 273:E99–E107. 9252485 10.1152/ajpendo.1997.273.1.E99

[phy213725-bib-0028] Reidy, P. T. , C. S. Fry , S. Igbinigie , R. R. Deer , K. Jennings , M. B. Cope , et al. 2017 Protein supplementation does not affect myogenic adaptations to resistance training. Med. Sci. Sports Exerc. 49:1197–1208. 28346813 10.1249/MSS.0000000000001224 PMC5433887

[phy213725-bib-0029] Shamim, B. , J. A. Hawley , and D. M. Camera . 2018 Protein availability and satellite cell dynamics in skeletal muscle. Sports Med., 48:1329–1343. 29557519 10.1007/s40279-018-0883-7

[phy213725-bib-0030] Shu, L. , and P. J. Houghton . 2009 The mTORC2 complex regulates terminal differentiation of C2C12 myoblasts. Mol. Cell. Biol. 29:4691–4700. 19564418 10.1128/MCB.00764-09 PMC2725723

[phy213725-bib-0031] Volpi, E. , H. Kobayashi , M. Sheffield‐Moore , B. Mittendorfer , and R. R. Wolfe . 2003 Essential amino acids are primarily responsible for the amino acid stimulation of muscle protein anabolism in healthy elderly adults. Am. J. Clin. Nutr. 78:250–258. 12885705 10.1093/ajcn/78.2.250 PMC3192452

[phy213725-bib-0032] Wang, Y. X. , and M. A. Rudnicki . 2011 Satellite cells, the engines of muscle repair. Nat. Rev. Mol. Cell Biol. 13:127–133. 22186952 10.1038/nrm3265

[phy213725-bib-0033] Winter, A. , and A. Bornemann . 1999 NCAM, vimentin and neonatal myosin heavy chain expression in human muscle diseases. Neuropathol. Appl. Neurobiol. 25:417–424. 10564532 10.1046/j.1365-2990.1999.00178.x

